# Diseased Meats

**Published:** 1875-07

**Authors:** 


### DISEASED MEATS.

There are some new arguments against the use of beef as an article of diet in certain localities near New York. The Farmers' Clubs in Union, Mercer, and Essex, and some of the other counties of New Jersey, have had their attention called to the unusual prevalence among the cattle, of a disease which has so far baffled all veterinary skill. It is known as pleuro pneumonia. It is accompanied by a running at the nostrils, a refusal of food, a gradual wasting away and in nine cases out of ten results in death. Cattle doctors have tried all the usual specifics for the disease, but have found them powerless to cure. Innoculation has been resorted to, but without effect. The most of such cattle have been pronounced, as might, indeed, reasonably be presumed, to be unfit for the market. Mortification follows almost immediately after death, and the stench emitted by the body of the dead animal is extremely offensive. In view of its prevalence, a law has been passed, the intent of which was to inflict a heavy penalty upon any and all parties instrumental in getting to the market such cattle as may have been seized with the malady, but the law has been discovered to be so defective that it is practically useless. In fact, those who have given the matter their attention say that the law practically operates as a premium to the concealment of the disease. Several of the farmer's clubs have nevertheless directed their officers to bring to the notice of the proper authorities, the cases of all persons who may be found engaged in the sale of diseased cattle. A number of farmers and butchers are reported to have sold and bought cattle so infected. The farmers have in some cases concealed the fact that the disease exists, from the butchers, who have innocently enough put it upon their stands for sale. On the other hand cases are reported in which the butcher is the guilty party. They go to the farmer and representing that they want the deceased carcas for the fat and tallow, dress them and dispose of them to the public. Vigorous measures have been resorted to to bring these cases to light, and to punish the guilty parties.

It is not speculation to suppose that the day will come when the use of flesh food will be largely abandoned and be superceded by the grains. It has been fully determined that human beings can live and thrive, and perform efficient service, alike with brain and hands, on a vegetable diet. The grains and fruit and vegetables supply all that is really demanded. Wheat alone is more valuable as food than sirloin steaks. If any argument could convert a meat-eater into a vegetarian, the traffic in diseased cattle will accomplish the result. A cattle-raiser of large experience informs us that by far the greater part of all beeves slaughtered, have some organic disease—either of heart or lungs or liver or other internal part. He is a skillful anatomist, and an educated gentleman, and knows whereof he affirms. These diseases are not ordinarily such as essentially affect the appearance of the animal.

It is reasonable to suppose that such food can not be eaten without doing serious injury to health.

---

UNSELFISHNESS.—There is a story of a young physician who, in the time of plague, arranged all his affairs—he had brilliant prospects—and took the care of a plague-stricken patient until he himself was seized. Then taking his watch and note-book, he marked down, stage by stage, the progress of the disease up to a point at which he could no longer hold the pen. Then he died. He did this that he might leave, through his suffering, knowledge that would save the lives of milions of men. Do you understand that? Yes, let us hope that there are still enough ingenious natures to realize and appreciate the heroism of that man's deed. There is another story of a man who went through all his life in humble circumstances, toiling and hoarding until he became known as a miser, and was jeered at and despised. He was no miser, however. He had an end in view. The poor of his city suffered greatly for the want of water. His object in amassing money was to build an aqueduct from the distant hills to provide the town with water. He died and bequeathed all the treasure he had accumulated to that purpose. He suffered poverty and humiliation without a murmer, that his fellow-citizens might not suffer from thirst. Are not these examples worthy of the consideration of every man?

---

Bounty, being free itself, thinks all others so.

